# Using high‐resolution array tomography to validate the use of synaptic vesicle glycoprotein 2A as a biomarker for synaptic loss in Alzheimer's disease

**DOI:** 10.1002/alz70856_104271

**Published:** 2025-12-26

**Authors:** Lauren Young, Declan King, Ya Yin Chang, Kristjan Holt, Scott Gladstein, Sjoerd J Finnema, Tara L. Spires‐Jones

**Affiliations:** ^1^ University of Edinburgh, Edinburgh, Scotland, United Kingdom; ^2^ Centre for Discovery Brain Sciences at the University of Edinburgh, Edinburgh, Scotland, United Kingdom; ^3^ The University of Edinburgh, Edinburgh, UK, United Kingdom; ^4^ UK Dementia Research Institute at the University of Edinburgh, Edinburgh, Scotland, United Kingdom; ^5^ Abbvie Inc., North Chicago, IL, USA; ^6^ Yale School of Medicine, New Haven, CT, USA

## Abstract

**Background:**

Numerous studies have used Synaptic Vesicle Glycoprotein 2A (SV2A) as a radioligand target during in‐vivo Positron Emission Tomography (PET) in humans to investigate the synaptic loss that strongly correlates with cognitive decline in Alzheimer's disease (AD). These studies showed a decrease in the SV2A PET signal in AD patient brains compared to controls highlighting the signals potential as a clinical biomarker. However, it is currently unclear whether this decreasing signal reflects synapse loss or a loss of SV2A from within the remaining synapses. This study uses array tomography, a high‐resolution imaging technique allowing quantification of synapse density and protein colocalisation, with the intent to validate whether SV2A could be used to accurately detect synapse loss in AD.

**Method:**

We compare SV2A puncta density, localisation and intensity with synaptophysin, a well characterised pre‐synaptic marker previously used to identify AD‐associated synapse loss, in human post‐mortem tissue samples from the inferior temporal gyrus, dorsolateral prefrontal cortex, entorhinal cortex, and cerebellum from end‐stage AD and age‐matched control subjects (*n* = 11‐19 cases per group for each brain region). Data are analysed with linear mixed effects models and Spearman's correlations.

**Result:**

We observe strong correlations between SV2A puncta density and synaptophysin puncta density within the same tissue samples, indicating SV2A is a reliable marker of synapse density. We further observe differences between brain regions in synapse density and in disease‐associated synapse changes. Preliminary analyses of SV2A intensity within synaptic puncta do not show differences between AD and control across regions indicating remaining synapses likely do not have altered levels of SV2A protein.

**Conclusion:**

These data indicate that changes in SV2A signal observed in AD with PET imaging likely reflect changes in synapse density rather than changes in protein levels within remaining synapses. Future work with the Foundations for the National Institutes of Health SV2A project team will integrate these data with in vivo PET imaging and molecular analyses of post‐mortem tissue to provide further understanding of the biological underpinnings of SV2A PET signal with the goal of determining whether SV2A PET will be a reliable marker of synapse density for use in future clinical use.

